# Gallstone Ileus: A Case Report of an Uncommon Cause of Mechanical Intestinal Obstruction

**DOI:** 10.7759/cureus.109649

**Published:** 2026-05-25

**Authors:** Oscar Alejandro Romero Arguello, Héctor Hernández Bonilla, Ulises Quintero Altamirano, Carlos Bess Oberto-Meraz, Gloria Isela Mendoza-Frías

**Affiliations:** 1 General Surgery, Hospital Universitario de Saltillo, Saltillo, MEX

**Keywords:** cholecystocolonic fistula presenting with gallstone ileus: a rare surgical case report, complicated acute cholecystitis, gallstone cholecystitis, intestinal obstruccion, rigler´s triad

## Abstract

Biliary ileus is an uncommon cause of mechanical intestinal obstruction secondary to the passage of gallstones into the gastrointestinal tract through a cholecystoenteric fistula. Its nonspecific clinical presentation contributes to diagnostic and therapeutic delays. Although computed tomography is considered the diagnostic modality of choice, identification of Rigler's triad on plain abdominal radiography may provide an early diagnostic orientation in patients with a compatible clinical presentation.

We present the case of a 58-year-old male patient without known biliary disease who presented with abdominal pain, bilious vomiting, and signs of intestinal obstruction. Plain abdominal radiography demonstrated pneumobilia, dilated bowel loops, and an ectopic gallstone at the ileocecal region, findings consistent with Rigler's triad. Given the high clinical and radiographic suspicion of biliary ileus, exploratory laparotomy was performed, revealing a gallstone impacted in the distal ileum. Isolated enterolithotomy was carried out with favorable postoperative outcomes.

This case highlights the importance of maintaining a high index of suspicion in patients presenting with subacute mechanical intestinal obstruction, even in the absence of documented biliary disease, in order to facilitate timely diagnosis and appropriate surgical management.

## Introduction

Gallstone ileus is defined as a mechanical intestinal obstruction secondary to the impaction of one or more large gallstones in the gastrointestinal tract. It was first described in 1654 by Thomas Bartholin, and decades later, Rigler identified the classic radiological findings that bear his name [1].

This is an uncommon complication of cholelithiasis, representing between 0.3% and 0.5% of all patients with gallstones and is one of the rarest causes of gallstone ileus, occurring in less than 0.1% of all cases of mechanical obstruction and 1% to 4% of non-strangulating mechanical obstructions of the small intestine [2]. Its incidence can increase significantly to 20% to 30% in women over 65 years of age [3], attributable to the higher prevalence of cholelithiasis in women and the increase in life expectancy. Mortality ranges from 2% to 25%, being higher than that of other causes of mechanical obstruction, related to advanced age, the presence of comorbidities, delayed diagnosis, and complications inherent to the obstructive condition [4].

Gallstone ileus is usually preceded by recurrent episodes of acute cholecystitis [5], chronic inflammation of the gallbladder caused by the impaction of a stone in the cystic duct, with subsequent obstruction of gallbladder drainage, and compromises arterial, venous, and lymphatic flow. This inflammatory process favors the formation of adhesions between the gallbladder and the gastrointestinal tract, which, added to the pressure exerted by the stones, causes ischemia and necrosis of the wall, facilitating erosion and the formation of a cholecystoenteric fistula. The cholecystoduodenal fistula is the most frequent, representing approximately 85% of cases. The remaining 15% are hepatoduodenal, choledochoduodenal, cholecystogastric, cholecystojejunal, and cholecystocolonic fistulas [3], which allow the passage of stones into the intestinal lumen [6]. In very rare cases, stones can pass directly into the duodenum through the common bile duct, following an endoscopic retrograde cholangiopancreatography (ERCP) or a sphincterotomy [7].

Although it is usually a single stone, up to 15% can be multiple [4,8]. A diameter greater than 2 cm is required to cause a mechanical intestinal obstruction; when this occurs, the most frequent site of impaction is at the level of the terminal ileum (60%), followed by the jejunum (30%) and colon (2.5%) [9].

The clinical presentation of gallstone ileus is usually nonspecific and of variable course, characterized by recurrent episodes of abdominal pain alternating with improvement, attributable to the retrograde displacement of the stone with a valvular effect, with subsequent progression to complete obstruction [5]. It includes colicky abdominal pain, distension, nausea and vomiting, hematemesis due to mucosal erosion, and diarrhea, which can lead to diagnostic confusion with gastroenteritis [3]. The average interval between the onset of symptoms and hospital admission is usually five to seven days, and the time to surgical intervention can extend to seven to 10 days [10].

Preoperative diagnosis remains a challenge for the surgeon; it should begin with an adequate medical history and physical examination, aimed at ruling out more frequent causes of intestinal obstruction, such as post-surgical adhesions, complicated hernias, or neoplasms.

Plain abdominal radiography is usually the first study ordered and can show signs of intestinal obstruction; however, its sensitivity is limited, reaching only 50%. The presence of Rigler's triad, consisting of pneumobilia, distended bowel loops with air-fluid levels, and an ectopic stone, is suggestive of the diagnosis, although it is not always present [3]. It has been shown that pneumobilia and radiographic signs of intestinal obstruction can be seen in up to 40% of cases, since most gallstones are radiolucent and cannot be identified. Several studies have indicated that Rigler's triad is present in only about 15% of patients, which limits its diagnostic utility in the early stages [1].

Currently, contrast-enhanced computed tomography is considered the method of choice, with an overall sensitivity, specificity, and diagnostic accuracy of 93%, 100%, and 99%, respectively. This has aided in diagnosis, allowing for precise identification of the stone's location, the site and level of obstruction, as well as the presence of pneumobilia and bilioenteric fistula [11,12]. However, the definitive diagnosis of this condition is established postoperatively in most cases. The treatment is surgical and its management continues to be a matter of controversy; the main objective is to resolve the intestinal obstruction, optimize the patient's general condition, correct hydroelectrolytic imbalances and manage comorbidities [13].

Isolated or simple enterolithotomy is the most commonly used procedure. It is associated with shorter surgical time and a lower complication rate; however, recurrence rates are estimated to be between 5% and 20% [14]. Single-stage surgery, which includes enterolithotomy, cholecystectomy, and fistula closure, is reserved for selected patients in good general condition. It is especially recommended in the presence of acute cholecystitis, gangrenous cholecystitis, or residual cholelithiasis [15,16]. Two-stage surgery, consisting of enterolithotomy alone followed by interval cholecystectomy and fistula repair, has been suggested for the treatment of gallstone ileus in young patients at risk of subsequent biliary complications. There is no consensus on the established time period between the first and second stage; however, it is normally performed after 4 to 6 weeks, although in some cases it may be deferred up to 6 months later [17].

An evaluation of the clinical approach and surgical management was conducted in a cohort of 12 patients with gallstone ileus between January 2018 and December 2024 at the Sancaktepe Şehit Prof. Dr. Ilhan Varank Training and Research Hospital in Istanbul. Isolated or simple enterolithotomy was performed in eight of the patients studied: six via open surgery and two via laparoscopy. Single-stage procedures were performed in the remaining four patients, using three laparoscopic and one open approaches. The choice of surgical treatment was individualized based on the risk factors identified in the study patients, including advanced age, the presence of comorbidities, and late presentation of the obstructive condition, all of which are associated with increased morbidity and mortality. The study concludes that the laparoscopic approach is feasible in hemodynamically stable and carefully selected patients, provided the surgeon has adequate experience. Otherwise, the open approach remains a safe and effective option [18].

Currently, there is no gold standard for the surgical management of gallstone ileus or for the treatment of its recurrences. However, the available evidence favors simple enterolithotomy, preferably using minimally invasive approaches when feasible, because it is associated with better postoperative outcomes and a lower complication rate.

## Case presentation

A 58-year-old male patient, with no history of chronic degenerative or surgical conditions, presented with a six-day history of colicky abdominal pain, rated 8/10 on the visual analog scale, of sudden onset, occurring at night, and located in the epigastrium, without initial radiation or alleviating factors. He subsequently experienced an exacerbation of the pain radiating to the mesogastrium, associated with decreased appetite, nausea, and multiple episodes of vomiting of bilious contents, as well as Bristol stools (type 7). He was evaluated twice by a physician, receiving symptomatic treatment without improvement, and therefore sought care at the emergency department of the University Hospital of Saltillo “Dr. Gonzalo Valdés Valdés”. His vital signs upon admission were as presented in Table 1.

**Table 1:** Vital signs recorded upon patient admission, results and reference ranges in adults.

Parameter	Result	Reference range (Adult)
Blood pressure	130/80 mmHg	120/80 mmHg
Heart rate	110 bpm	60-100 beats per minute (bpm)
Respiratory rate	20 breaths/min	12-20 breaths per minute (breaths/min)
Temperature	37.5°C=99.5 °F	36.0-37.5°C (96.8-99.5°F)
Oxygen saturation	95% SpO₂	95%-100% (SpO₂) normal oxygen saturation

Physical examination revealed a pain-related facial expression, dehydration of the mucous membranes, a distended abdomen due to adipose tissue, decreased peristalsis in frequency and intensity in the left hemiabdomen, absent in the right hemiabdomen, pain on deep palpation in all four quadrants with hyperalgesia, and signs of generalized peritoneal irritation. Paraclinical tests were ordered, and the parameters are presented in Table 2.

**Table 2:** Laboratory results upon patient admission Only altered parameters are recorded, and the rest are within normal ranges.

Parameter	Result	Reference range (adult)
White blood cells	18.69x10³/µL	4.0-10.0x10³/µL
Neutrophils (%)	89%	40%-70%
Creatine	1.7 mg/dl	0.6-1.3 mg/dl
Estimated glomerular filtration rate (EFGR)	47 mL/min	>90 mL/min
Total bilirubin	2.18 mg/dl.	0.3-1.2 mg/dl
Direct bilirubin	0.76 mg/dl	0.0-0.3 mg/dl
Indirect bilirubin	1.42 mg/dl	0.2-0.8 mg/dl
Gamma-glutamyl transferase (GGT)	122 U/l	9-48 U/l (lab-dependent)

**Figure 1:**
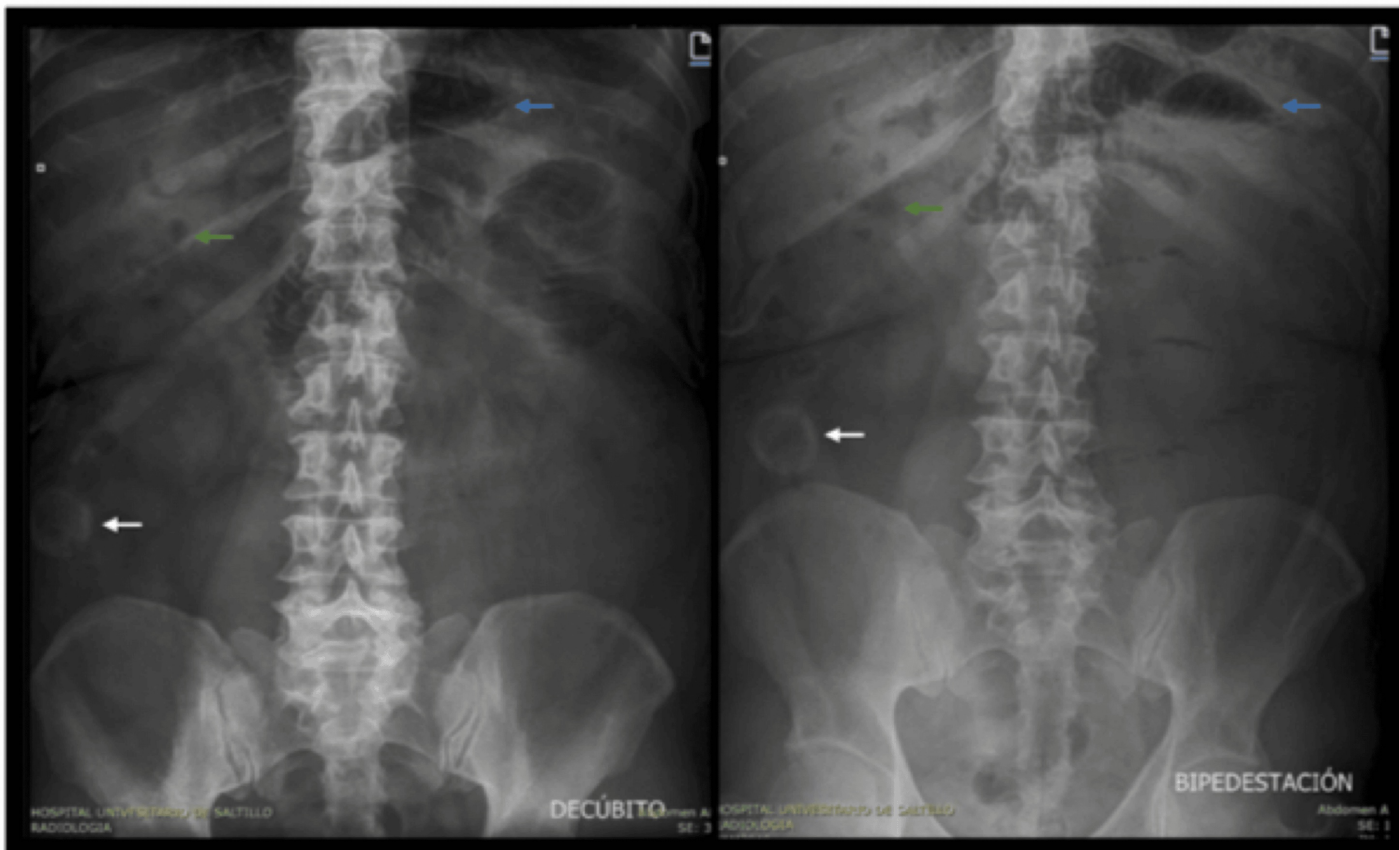
Plain abdominal radiograph in two positions. Green arrow: pneumobilia, blue arrow: dilation of intestinal loops, air-fluid levels, white arrow: gallstone.

**Figure 2:**
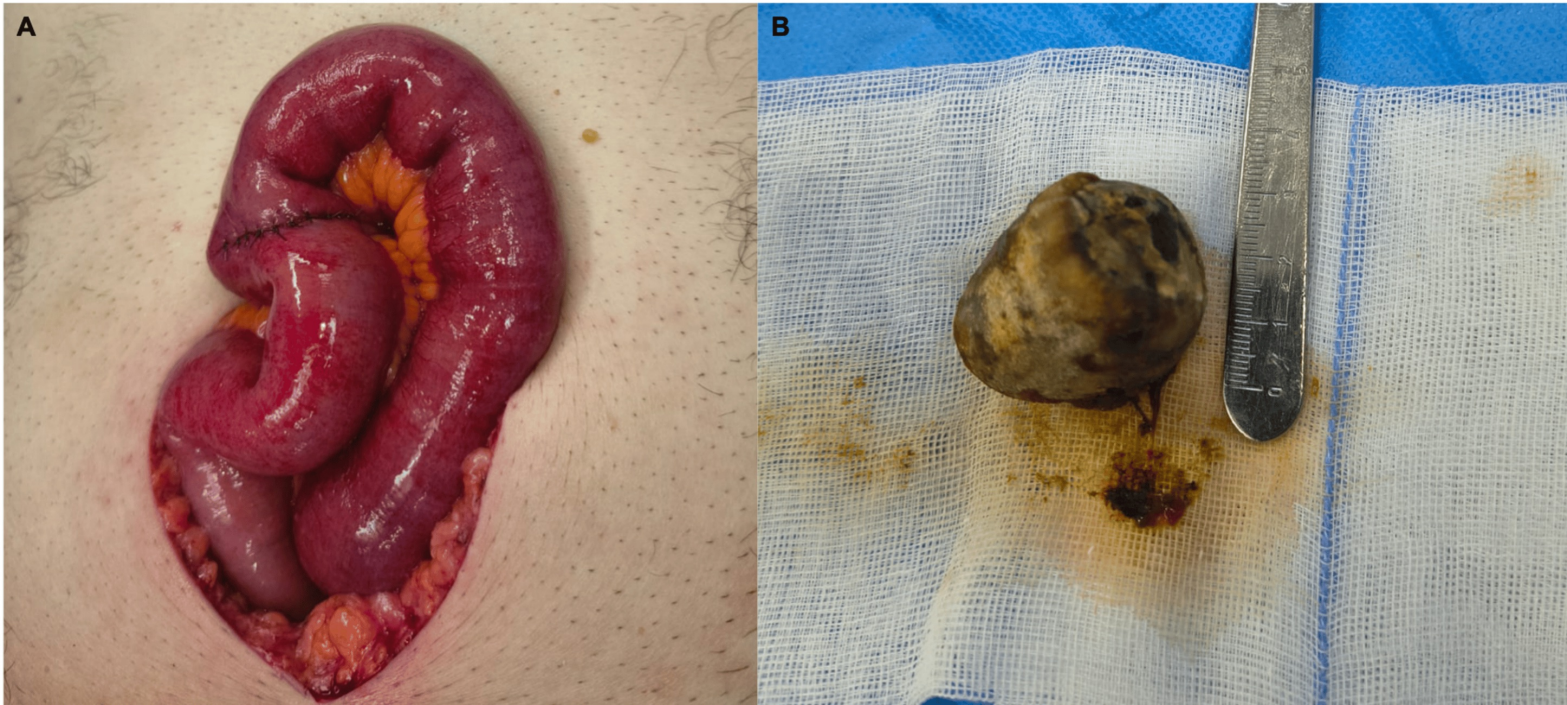
Intraoperative findings: A: enterolithotomy, B: 4 cm stone extracted.

A plain abdominal radiograph in anteroposterior projection was requested, showing dilation of small bowel loops and air-fluid levels, pneumobilia, and a well-defined radiopaque image located at the level of the ileocecal valve, consistent with Rigler's triad (Figure 1). Due to the presence of signs of peritoneal irritation, a high suspicion of clinical and radiographic gallstone ileus, and the unavailability of computed tomography, the patient was scheduled for exploratory laparotomy. After admission to the operating room, under balanced general anesthesia, in the supine position, after skin preparation with a chlorhexidine-based antiseptic solution and placement of sterile drapes, a midline infraumbilical incision was made to access the abdominal cavity. Dilation of small bowel loops and a transition point at the level of the distal ileum were identified. A stone approximately 4 cm in diameter was manually located, impacted 20 cm from the ileocecal valve (Figure 2). An isolated longitudinal enterotomy was performed along the antimesenteric border, with extraction of the stone and primary closure in two layers using absorbable sutures (Heineke-Mikulicz type). A bilioenteric fistula was also identified, which was not addressed during this surgical procedure. No abdominal drains were placed. Adequate hemostasis was verified, a complete count of textile materials and instruments was taken, and the abdominal wall was closed in layers.

The patient was hemodynamically stable in the immediate postoperative period and a progressive diet was initiated after the return of bowel function. He was discharged on the third postoperative day with instructions for monitoring of the surgical wound and suture removal in seven to 10 days at an outpatient clinic. The procedure performed corresponds to a standard surgical technique widely described in the literature, without the use of novel devices.

## Discussion

Gallstone ileus continues to pose a diagnostic and therapeutic challenge due to its low incidence, nonspecific clinical presentation, and frequent late diagnosis. Although computed tomography is the imaging study of choice, the identification of Rigler's triad on plain abdominal radiography, described in approximately 15% of cases, along with a compatible clinical presentation, can guide the diagnosis and allow for timely surgical treatment.

While this condition is more common in elderly women with a history of cholelithiasis, this case demonstrates that it can also occur in male patients without a prior diagnosis of biliary disease. It has been reported that up to 50% of patients with gallstone ileus have no known history of cholelithiasis [6], which contributes to diagnostic delays and multiple symptomatic treatments before definitive hospital admission. Therefore, gallstone ileus should be considered in the differential diagnosis of mechanical intestinal obstruction, even in the absence of a documented biliary history.

In the present case, isolated enterolithotomy was performed through exploratory laparotomy. This decision was based on the prolonged clinical course, marked abdominal distension, and the need for prompt surgical resolution with lower operative complexity. Furthermore, the open approach allowed adequate localization of the obstructive site and safe extraction of the gallstone. Repair of the cholecystoenteric fistula was not performed and was deferred to a second-stage procedure. It is important to highlight that spontaneous closure of the cholecystoenteric fistula has been reported in more than 50% of cases during postoperative follow-up after isolated enterolithotomy for gallstone ileus [19].

This procedure remains a feasible and widely accepted therapeutic strategy in the literature and international surgical guidelines for the management of gallstone ileus.

Although laparoscopic approaches represent an attractive minimally invasive alternative, patient selection should be individualized according to clinical status, duration of symptoms, associated comorbidities, and technical feasibility, with the aim of ensuring a safe and effective resolution of the obstructive condition [20].

Surgical decision-making should be based on a comprehensive and individualized assessment, considering both the available evidence and the experience of each hospital. A high index of clinical suspicion and timely recognition of gallstone ileus allow for early treatment and can potentially reduce the morbidity and mortality associated with this rare but clinically relevant condition.

## Conclusions

Gallstone ileus continues to represent a diagnostic and therapeutic challenge due to its low incidence and nonspecific clinical presentation, factors that contribute to delayed diagnosis and increased associated morbidity and mortality. The present case highlights the importance of maintaining a high index of suspicion in patients presenting with mechanical intestinal obstruction, even in the absence of documented biliary disease.

Although computed tomography remains the diagnostic modality of choice, identification of Rigler’s triad on plain abdominal radiography, together with a compatible clinical presentation, may facilitate early diagnosis and justify timely surgical intervention. In this context, resolution of the obstructive process remains the primary therapeutic objective through individualized strategies, including isolated enterolithotomy, one-stage surgery, or delayed management of the cholecystoenteric fistula.

The selection of the surgical approach, whether open or laparoscopic, should be based on the patient’s clinical and hemodynamic status, associated comorbidities, duration of symptoms, and the experience of the surgical team. Therefore, appropriate clinicoradiological integration and individualized decision-making are essential to optimize clinical outcomes in this uncommon entity.
